# Opposite Fates Under Warming: Climatic Suitability, Niche Divergence and Phenological Exposure of *Riptortus pedestris* and *Nezara viridula* in Soybean

**DOI:** 10.3390/insects17080753

**Published:** 2026-07-23

**Authors:** Mingyang Zou, Xueyan Zhang, Ai Xia

**Affiliations:** 1 State Key Laboratory of Agricultural and Forestry Biosecurity, College of Plant Protection, Nanjing Agricultural University, Nanjing 210095, China; 2024802157@stu.njau.edu.cn (M.Z.); 2022202022@stu.njau.edu.cn (X.Z.); 2 Key Laboratory of Soybean Disease and Pest Control, Ministry of Agriculture and Rural Affairs, College of Plant Protection, Nanjing Agricultural University, Nanjing 210095, China

**Keywords:** *Riptortus pedestris*, *Nezara viridula*, MaxEnt, climatic niche, phenological matching, soybean

## Abstract

The bean bug, *Riptortus pedestris* (Fabricius), and the southern green stink bug, *Nezara viridula* (L.), are important pod-sucking pests of soybean, *Glycine max* (L.) Merr. Their feeding on pods and developing seeds induces soybean staygreen symptoms and causes severe yield losses. To assess their future damage risk, we integrated MaxEnt, PCA env and a phenological matching index (PMI) using global occurrence records, bioclimatic variables and soybean phenology data. Under current climatic conditions, *R. pedestris* exhibited a predominantly temperate East Asian distribution, with suitable areas extending farther north and northeast. By contrast, *N. viridula* showed a more southerly and spatially continuous distribution across South and Southeast Asia, with suitability declining markedly toward northern and northeastern Asia. By the 2090s under SSP5-8.5, suitable areas decreased by 26.7% for *N. viridula* but increased by 34.0% for *R. pedestris*, and the co-suitable area declined from 9.460 to 7.350 million km^2^. PCA env indicated low to moderate niche overlap (Schoener’s D = 0.278) with significant niche differentiation. Under fixed soybean calendars, mean PMI generally increased for both pests, although the increase plateaued for *R. pedestris* under high emission scenarios by the late 21st century. Collectively, these findings indicate that Asian soybean production regions currently face overlapping damage risk, that the potential damage zone of *R. pedestris* is likely to expand further, and that the temporal overlap between the soybean sensitive period and adult activity windows of both pests will generally increase—despite the projected contraction in the potential damage distribution of *N. viridula*.

## Simple Summary

*Riptortus pedestris* and *Nezara viridula* are major pod-feeding pests of soybean in East Asia. By feeding on pods and developing seeds during critical soybean reproductive stages, both species induce staygreen syndrome, leading to substantial yield losses. Under future climate warming, both the climatically suitable habitats of these pests and the temporal synchrony between the soybean sensitive period and adult activity windows are projected to shift. Here, we used MaxEnt to model the potential geographic distributions of both species and a phenological matching index to quantify their phenological overlap with soybean. Our objectives were to assess their future damage risk across East Asian production regions and to inform proactive early warning and management strategies.

## 1. Introduction

Soybean (*Glycine max* (L.) Merr.) is one of the world’s most economically important oilseed and protein crops [1]. Among the major pest taxa of soybean, true bugs (Hemiptera: Heteroptera) are particularly damaging during reproductive growth, as their feeding on developing pods and seeds directly compromises both yield quantity and seed quality [2,3,4,5]. Global warming is expected to continue during the 21st century [6]. With climate change expected to alter the diversity and distribution of soybean true bugs [7], there is a need to assess how the potential damage risk of key pod-sucking pests may change in major soybean production regions.

To date, climate change impacts on the potential worldwide distribution have been modeled for *Piezodorus guildinii* (Hemiptera: Pentatomidae) [8]. By contrast, two other major soybean pests, the bean bug *Riptortus pedestris* (Fabricius) (Alydidae) and the southern green stink bug *Nezara viridula* (L.) (Pentatomidae), have received considerably less attention in comparative climatic analysis. The two species show partially overlapping geographic distributions in major Asian soybean-growing regions: *R. pedestris* has been reported from China, Japan, India, Korea, and Vietnam, while *N. viridula* has also been recorded in China, Japan and India and extends more broadly to countries such as the United States and Brazil [9,10]. Existing evidence indicates that the northern distributional limit of *N. viridula* in Japan has expanded poleward in response to winter warming and relaxed overwintering constraints [11,12]. However, a marked decline in the relative abundance of *N. viridula* has also been reported in the Neotropical region [13]. By contrast, MaxEnt-based projections suggest that *R. pedestris* will expand its climatically suitable range under future climate scenarios, both at its northern range margin and in tropical regions [9]. Whether the two pests will show different changes in climatically suitable areas under future climate warming remains unclear. Therefore, a direct comparison of the two species under a common analytical framework is needed [14].

Both *R. pedestris* and *N. viridula* are important pod-sucking pests of soybean. During soybean reproductive growth, they feed on pods and developing seeds, disrupting seed development, reducing seed quality and yield, and inducing delayed maturity or staygreen symptoms [2,3,4,5,15,16,17]. The period spanning from beginning pod to seed maturation (R3–R7) represents the critical window during which both species actively feed, and thus constitutes the primary phase of crop vulnerability to these pests [18].

Despite these advances, two fundamental questions remain unresolved: (1) how future climate warming will reshape the climatically suitable areas of *R. pedestris* and *N. viridula*; and (2) how to quantify, in a spatially explicit manner, the temporal overlap between adult activity windows and susceptible soybean stages. To address this knowledgeable gap, we (i) modeled current and future climatically suitable areas for both species and quantified their expansion, contraction and stability under future climate scenarios; (ii) evaluated climatic niche differentiation between the two species and quantified the reorganization of co-suitable, *R. pedestris*-only and *N. viridula*-only areas; and (iii) developed a Phenological Matching Index (PMI) to estimate the temporal overlap between potential adult activity windows and soybean R3–R7 stages within current soybean-growing areas, including the effects of discrete sowing-date adjustments.

## 2. Materials and Methods

### 2.1. Occurrence Records

Occurrence records of *R. pedestris* and *N. viridula* were compiled from GBIF downloads, field surveys, and georeferenced literature records (Table S1a); The GBIF records were obtained from two occurrence downloads: GBIF.org (23 March 2026) GBIF Occurrence Download https://doi.org/10.15468/dl.9prazt (accessed on 1 May 2026); GBIF.org (23 March 2026) GBIF Occurrence Download https://doi.org/10.15468/dl.uwm2kv (accessed on 1 May 2026).

All records were standardized to WGS84 decimal degree coordinates. Records lacking geographic coordinates, records with invalid coordinates, records located in the ocean, and records from which environmental values could not be extracted were removed. All analyses were conducted in R version 4.5.2. The R package Coordinate Cleaner (version 3.0.1) was then used to remove likely georeferencing errors, including records assigned to country capitals, administrative centroids, biodiversity institutions, zero coordinates, equal latitude and longitude values, and spatial outliers [19].

To reduce spatial sampling bias and match the spatial resolution of the environmental layers, occurrence records were spatially filtered to retain only one record per 2.5 arc-minute WorldClim grid cell using the R package terra (version 1.8.80). After coordinate cleaning, spatial filtering, and environmental extraction, 1985 records of *R. pedestris* and 21,160 records of *N. viridula* were retained for MaxEnt modeling. Mapping the cleaned occurrence records at a global scale showed that *N. viridula* was widely distributed worldwide, whereas the retained records of *R. pedestris* were restricted mainly to Asia (Figure S5). We therefore used global occurrence records and global climatic data to model both species, allowing their climatic associations to be characterized within a consistent global framework. Subsequent regional analyses focused on the Asian extent, where the distributions of the two species overlap, to facilitate a clearer comparison of their spatial patterns.

### 2.2. Climatic Data

Current climatic data were obtained from WorldClim v2.1 at 2.5 arc minute resolution and represented the baseline period 1970–2000 [20]. The 19 bioclimatic variables (BIO1–BIO19) were initially considered as candidate predictors for species distribution modeling. Monthly maximum and minimum temperature layers from WorldClim were also obtained at the same resolution for the phenological matching analysis.

Future climatic data were obtained from CMIP6 WorldClim projections for two future periods, the 2050s (2041–2060) and the 2090s (2081–2100), under four Shared Socioeconomic Pathway scenarios [21]: SSP1-2.6 (sustainability, approximately 1.8 °C warming by 2100), SSP2-4.5 (middle of the road, approximately 2.7 °C), SSP3-7.0 (regional rivalry, approximately 3.6 °C) and SSP5-8.5 (fossil fueled development, approximately 4.4 °C). Three general circulation models were used: BCC-CSM2-MR, IPSL-CM6A-LR, and MRI-ESM2-0. These models were selected because they provide both bioclimatic variables for MaxEnt modeling and monthly temperature variables for PMI calculation.

Species distribution models were calibrated and projected globally using global occurrence records and climatic data. Regional mapping and all area-based analyses were then restricted to a fixed Asian extent (60° E–150° E, 10° S–55° N), including suitable-area summaries, transition analysis, and two-species overlap analysis. This extent encompasses the principal East Asian soybean-producing regions and adjacent areas relevant to the projected redistribution of climatically suitable areas. It was used only for regional post-processing and did not affect global model calibration or projection.

Future SDM predictions and PMI calculations were conducted separately for each GCM, SSP and period. For each SSP and period combination, suitability outputs and PMI values from the three GCMs were then averaged. Raster geometry was checked before spatial overlay to avoid area or overlap bias.

### 2.3. Predictor Selection

To reduce multicollinearity among the 19 bioclimatic variables, predictor screening was conducted separately for each species before MaxEnt modeling. Pairwise Pearson correlation coefficients among the bioclimatic variables were first calculated based on the occurrence data of each species and the global bioclimatic data. When two variables were highly correlated (|r| > 0.7), one variable was removed, with priority given to retaining variables related to thermal variation, temperature extremes, and seasonal moisture conditions relevant to pest development and host-plant growth. Variance inflation factors (VIFs) were then calculated for the remaining variables, and variables with VIF > 10 were sequentially removed until all retained variables satisfied VIF ≤ 10 [22].

The final predictor set for *R. pedestris* included BIO18 (precipitation of the warmest quarter), BIO5 (maximum temperature of the warmest month), BIO14 (precipitation of the driest month), BIO2 (mean diurnal temperature range), BIO3 (isothermality) and BIO12 (annual precipitation). The final predictor set for *N. viridula* included BIO6 (minimum temperature of the coldest month), BIO5, BIO14, BIO18, BIO2, BIO15 (precipitation seasonality), BIO19 (precipitation of the coldest quarter), BIO13 (precipitation of the wettest month), BIO8 (mean temperature of the wettest quarter) and BIO9 (mean temperature of the driest quarter). The maximum VIF and pairwise |r| were 7.603 and 0.635 for *R. pedestris*, and 5.409 and 0.692 for *N. viridula*, respectively (Table S1c; Figure S1).

### 2.4. MaxEnt Model Selection and Setting

Climatic suitability was modeled using MaxEnt presence-background models [23,24,25], implemented with the R packages maxnet (version 0.1.4) [26] and ENMeval (version 2.0.5.2) [27]. A total of 10,000 global terrestrial background points with complete environmental values were randomly sampled using set.seed(42) [25,28,29]. The same background set was used for both species to provide a common environmental reference for cross-species comparison. Model tuning and final model fitting were conducted separately for the two species using their respective predictor sets.

Model calibration was performed with spatial block partitioning in ENMeval, which divided occurrence records into four spatial folds and assigned background points to corresponding blocks [27]. During model tuning, occurrence records were subsampled to a maximum of 1000 records per species. Final models were then refitted using all cleaned occurrence records and the 10,000 background points.

Five feature class combinations were tested: L, LQ, LQH, LQHP and LQHPT, where L, Q, H, P and T represent linear, quadratic, hinge, product and threshold features, respectively. Regularization multipliers ranged from 0.5 to 3.0 at intervals of 0.5, resulting in 30 candidate models for each species. The optimal model was selected according to the lowest corrected Akaike information criterion (AICc).

Model performance was evaluated using the area under the receiver operating characteristic curve (AUC), the true skill statistic (TSS) [30] and the continuous Boyce index (CBI) [31,32,33]. Variable importance was assessed by permutation importance, calculated as the normalized decrease in the model AUC after randomly permuting each predictor while keeping the other predictors unchanged. Final models were projected to current and future climate layers to generate climatic suitability maps for subsequent area and overlap analyses.

### 2.5. Model Evaluation and Suitability Analysis

Separate species-specific fixed cumulative value 5 (FCV5) thresholds were determined for *R. pedestris* and *N. viridula* and used to distinguish suitable from unsuitable areas for each species [24,34,35]. Grid cells with suitability values below the FCV5 threshold were classified as unsuitable, whereas values equal to or above this threshold were divided into low-, moderate-, and high-suitability classes using the Jenks natural breaks method. The species-specific FCV5 thresholds and Jenks class boundaries derived under current climatic conditions were held constant across all future scenarios.

Suitable areas were calculated using the cellSize() function in the R package terra (version 1.8.80) and summarized in million km^2^. Future changes were categorized by comparing current and future suitable grid cells. Stable areas were suitable in both the current and future periods; contraction areas were suitable only in the current period; and expansion areas were suitable only in the future period.

To characterize spatial overlap between the two species, suitable grid cells were assigned to three categories: *R. pedestris*-only, *N. viridula*-only, and co-suitable. Co-suitable areas were defined as areas suitable for both species.

### 2.6. Climatic Niche Analysis

Climatic niche differentiation between *R. pedestris* and *N. viridula* was analyzed using PCA env [36]. For PCA-env, a common predictor set was selected from BIO1–BIO19 by screening the occurrence-environment matrices of both species using the Pearson correlation and VIF criteria described in Section 2.3. Variables retained in the common set were required to satisfy |r| ≤ 0.7 and VIF ≤ 10 for both species. The resulting predictor set included BIO2, BIO5, BIO6, BIO14 and BIO18. Principal component analysis was first performed on the background climate space, and occurrence records of the two species were then projected into this environmental space using the R packages ecospat (version 4.1.2) and ade4 (version 1.7.23).

Niche densities were estimated on a 100 × 100 environmental grid. Niche overlap was quantified using Schoener’s D and Warren’s I [37,38]. One *N. viridula* occurrence record falling outside the background PCA space was excluded from the density calculation.

Niche equivalency and similarity tests were conducted with 1000 randomizations in ecospat. The equivalency test evaluated whether the two species occupied equivalent climatic niches, whereas the similarity test evaluated whether the observed niche overlap was greater than expected from the available background environments. Dynamic niche indices, including expansion, stability, and unfilling, were calculated with each species alternately used as the reference.

Sample size sensitivity was evaluated by randomly subsampling *N. viridula* records to match the number of *R. pedestris* records (*n* = 1985) and repeating the PCA env analysis 100 times. The resulting distributions of Schoener’s D and Warren’s I were compared with the full dataset estimates and are reported in Table S4.

### 2.7. Phenological Matching Index

We developed a phenological matching index (PMI) to quantify temporal overlap between potential pest adult activity and soybean R3–R7 stages within current cultivation areas. SPAM 2020 v2.0 Release 2 soybean physical area was used as a mask, retaining cells with soybean area >1 ha [39]. Soybean sowing and harvest dates came from the Sacks crop calendar [40].

The soybean R3–R7 window was approximated as 50% to 90% of the local sowing to harvest season. This approximation follows the general timing of soybean reproductive development: R3 (beginning pod) occurs near the middle of the growing season, and R7 (beginning maturity) precedes harvest [18]. The start and end of this crop-sensitive window were calculated separately for each soybean grid cell.

Potential adult activity windows were estimated from WorldClim monthly maximum/minimum temperatures using a sine wave degree day method [41]. Adults were selected as the temporal target because both pests can disperse into soybean fields and feed on pods or seeds, and adult feeding effects on pods, seed quality, and yield have experimental support [2,3,4].

Adult active windows were positioned using an effective degree day (DD) onset. For each grid cell, the local effective DD onset was the first monthly time step with positive accumulated growing degree days (GDD) under the species-specific lower developmental threshold (Tbase). Cumulative GDD was then tracked from this onset, and three potential adult-active windows were placed where cumulative GDD reached 1 K, 2 K, and 3 K, representing successive potential generations. Each window was 30 d (25 d and 35 d tested for sensitivity). Degree-day parameters were Tbase = 14.1 °C and K = 336.7 DD for *R. pedestris* [42] and Tbase = 13.4 °C and K = 434.8 DD for *N. viridula* [43].

No separate overwintered-adult onset temperature threshold or fixed pre-oviposition delay was applied. Adult-active windows were positioned directly from cumulative developmental degree-days. The immature-development thermal parameters are summarized in Table S5d.

$$O_{i}=\frac{max\left\{0,\;min\left(C_{e},\;F_{i}+w\right)-max\left(C_{s},\;F_{i}\right)\right\}}{w},i=1,\;2,\;3$$

$$PMI=\sum_{i=1}^{3}O_{i}=O_{1}+O_{2}+O_{3}$$

where *C_s_* and *C_e_* are the start and end day-of-year of the soybean R3–R7 window, *F_i_* is the start day of the *i*-th adult-active window (the day cumulative GDD from local effective-DD onset first reaches *i*K), and *w* is the window length (30 d baseline).

PMI ranges theoretically from 0 to 3. PMI = 0 denotes no overlap between the three potential adult-active windows and soybean R3–R7, whereas a value of 1 corresponds to the cumulative overlap of one full 30 d adult window. Higher PMI values denote stronger synchrony between potential adult activity and soybean-sensitive stages and can identify phenological exposure hotspots.

### 2.8. Sowing-Calendar Scenarios and Statistical Tests

Future PMI was calculated under fixed crop calendars, which combined future temperatures with current sowing and harvest dates, and under sowing-date adjustment scenarios (−30, −15, +15 and +30 d, with growing-season length unchanged). For each species, SSP and period, the adjustment yielding the lowest mean PMI among the four discrete scenarios was identified as the minimum-overlap scenario.

Grid-level PMI within the soybean mask was aggregated to the mean PMI of 5° × 5° spatial blocks to reduce spatial pseudo-replication, so that each block contributed a single mean-PMI value per scenario. For each comparison, the paired block means were tested with Wilcoxon signed-rank tests, and *p* values were adjusted using the Benjamini–Hochberg method [44].

We evaluated sensitivity to adult-window length using 25 d and 35 d scenarios around the 30 d baseline. Spearman rank correlation compared continuous PMI rankings between window lengths (Table S5c).

## 3. Results

### 3.1. Model Performance and Key Predictors

Both optimal models, trained on current climate data (1970–2000), performed well (Table 1; Figure S2; Table S2a). For *R. pedestris*, the best model was LQHPT, RM = 2.0, with AUC = 0.980, TSS = 0.912 and CBI = 0.820. For *N. viridula*, the best model was LQHP, RM = 0.5, with AUC = 0.958, TSS = 0.835 and CBI = 0.953. The species-specific FCV5 thresholds used for suitability classification were 0.023 for *R. pedestris* and 0.098 for *N. viridula*.

Permutation importance differed markedly between species. BIO18 dominated the *R. pedestris* model (63.3%), followed by BIO3 (20.1%) and BIO5 (12.0%). BIO6 dominated the *N. viridula* model (66.6%), followed by BIO5 (11.7%), BIO13 (8.1%) and BIO19 (6.7%). Response curves showed that *R. pedestris* suitability increased sharply as BIO18 increased from approximately 250 to 500 mm and then leveled off. For *N. viridula*, suitability was highest at intermediate BIO6 values around 0–10 °C and declined toward both colder and warmer conditions. Suitability also increased with BIO5 to a peak at approximately 25–30 °C and then declined at higher temperatures.

### 3.2. Current Climatic Suitability

**Figure 1:**
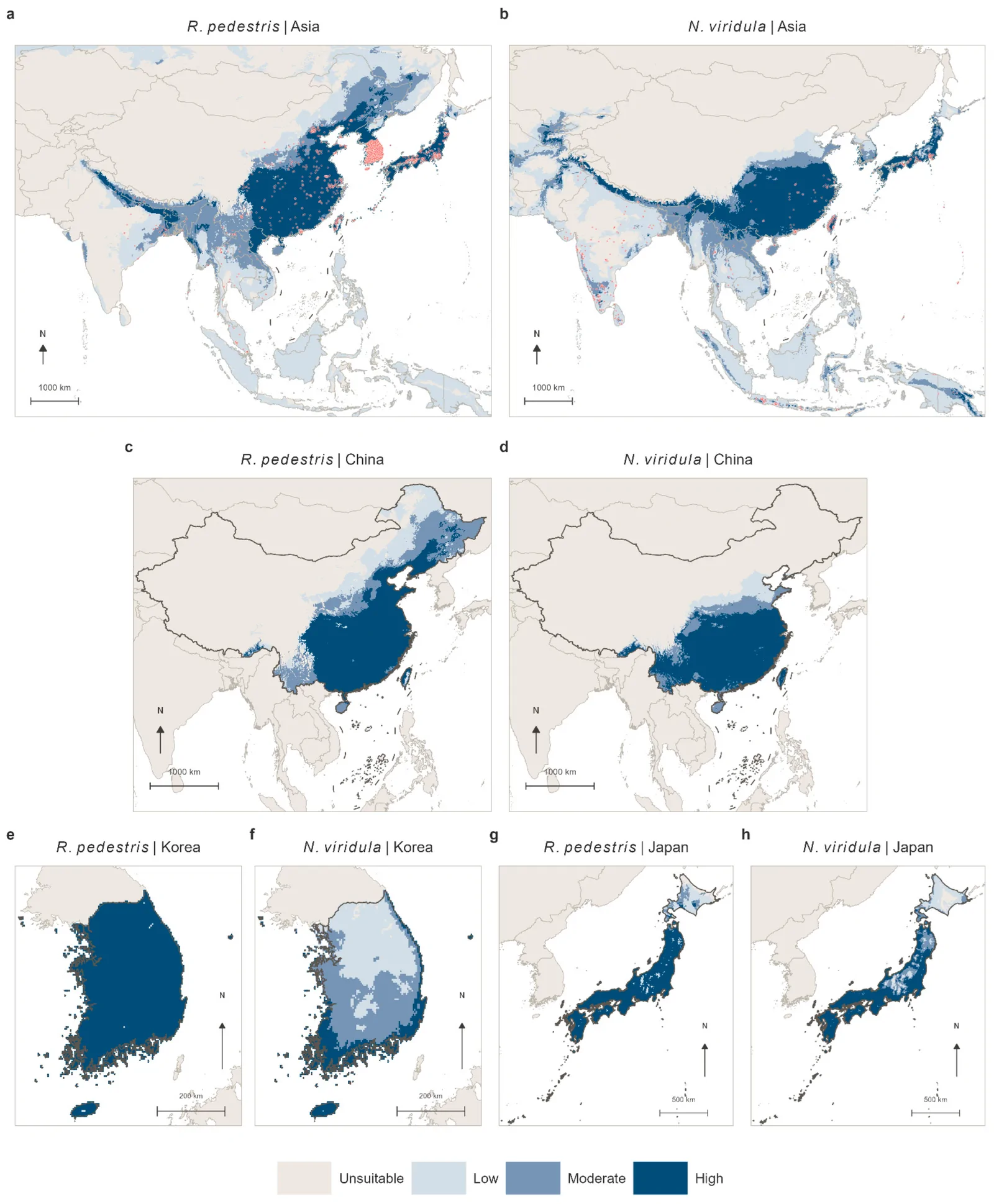
Current climatic suitability of *Riptortus pedestris* (**a**) and *Nezara viridula* (**b**) with occurrence records used for model fitting within the Asian analysis extent (60° E–150° E, 10° S–55° N). Inset panels show suitability cropped to national boundaries for China, South Korea and Japan: *R. pedestris* (**c**,**e**,**g**) and *N. viridula* (**d**,**f**,**h**). Occurrence points are displayed only on the full-extent panels.

Under current climatic conditions, the two species showed clearly contrasting latitudinal patterns within the Asian analysis extent (Figure 1; Table 2). *R. pedestris* had a more northerly distribution centered on temperate East Asia. Its high-suitability areas formed a broad core across central and eastern China, the Korean Peninsula and Japan, and extended farther into northern and northeastern China. At lower latitudes, its suitable areas were less continuous and were dominated by low- and moderate-suitability classes. In contrast, *N. viridula* showed a distinctly more southerly distribution. Its suitable areas formed a more continuous belt across southern China, South Asia and Southeast Asia, but became markedly restricted toward northern and northeastern China.

The regional panels further highlighted these differences. In China, high suitability for *R. pedestris* extended farther into the northern and northeastern regions, whereas high suitability for *N. viridula* was concentrated mainly in the southern, central and eastern regions. Most of South Korea was highly suitable for *R. pedestris*, while *N. viridula* was dominated by low- and moderate-suitability classes, with high suitability restricted mainly to the south. The contrast was weaker in Japan, where both species showed high suitability across much of the archipelago, with suitability decreasing toward Hokkaido.

The total suitable area was 12.599 million km^2^ for *R. pedestris* and 12.430 million km^2^ for *N. viridula*. For *R. pedestris*, low-, moderate- and high-suitability areas covered 6.456, 2.663 and 3.481 million km^2^, respectively. The corresponding areas for *N. viridula* were 6.755, 2.659 and 3.016 million km^2^. Most cleaned occurrence records displayed in the Asian overview panels fell within the predicted suitable areas of the corresponding species.

### 3.3. Future Changes in the Spatial Distribution of Suitable Areas

**Figure 2:**
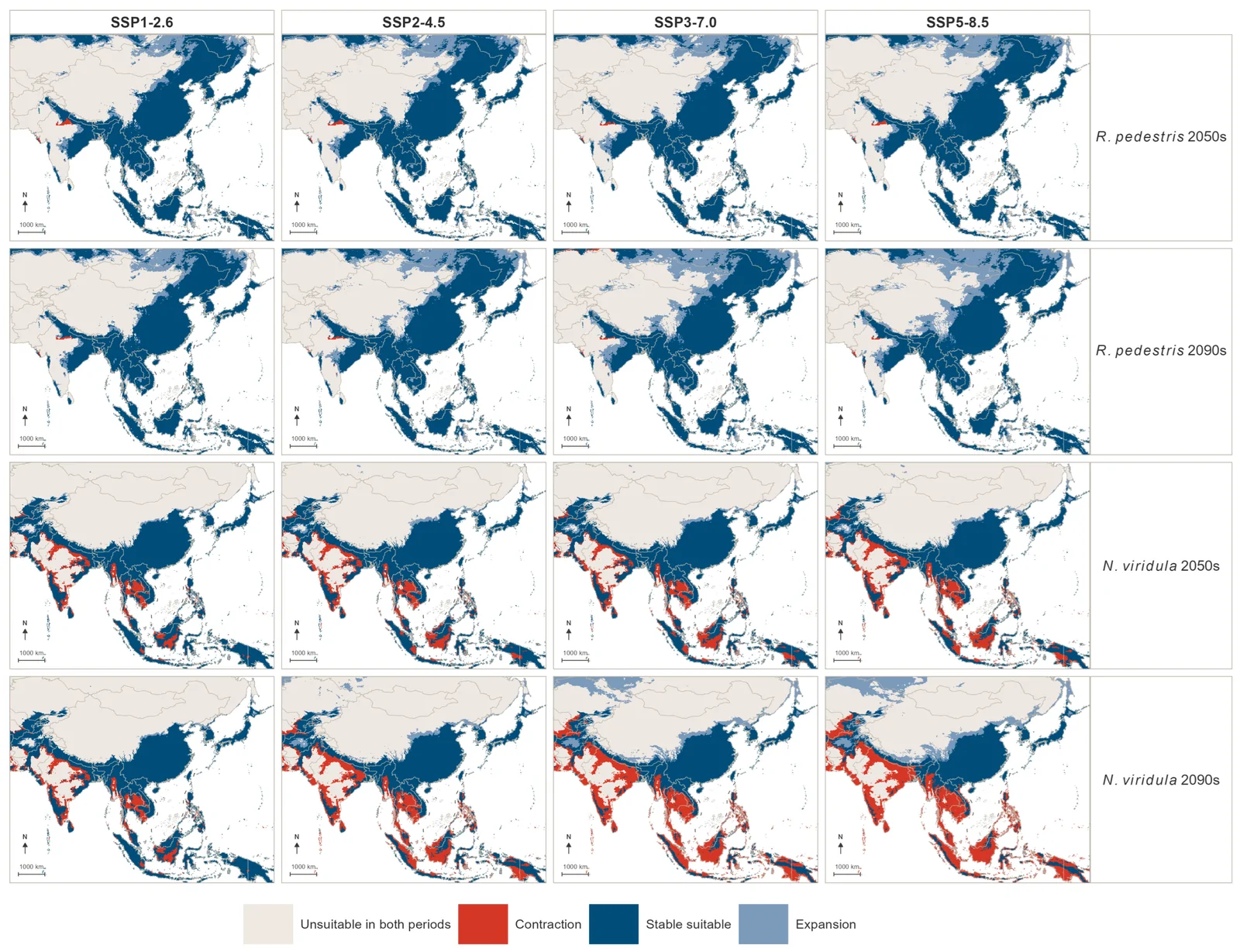
Suitable-area transitions (contraction, stability and expansion) for *R. pedestris* and *N. viridula* under future SSP scenarios relative to current climatic suitability.

Future projections revealed contrasting spatial changes in the climatically suitable areas of the two species within the Asian analysis extent (Figure 2; Figure S4; Table 2 and Table S3). For *R. pedestris*, large portions of the current suitable areas across central and eastern China, the Korean Peninsula, and Japan remained stable across future scenarios. Expansion occurred mainly from northern and northeastern China across Mongolia to southern Siberia and the Russian Far East. Additional newly suitable areas appeared in northwestern China, adjacent parts of Central Asia, and scattered parts of South Asia. These expansion areas were already apparent in the 2050s and became more extensive by the 2090s, particularly under SSP3-7.0 and SSP5-8.5. Contraction remained limited and occurred mainly as small, scattered patches around the northwestern Indian subcontinent and the Himalayan foothills.

*N. viridula* showed a different spatial trajectory. Stable suitable areas were retained mainly in central and eastern China, the Korean Peninsula and Japan. Extensive contraction occurred across the Indian subcontinent, mainland Southeast Asia, the Malay Peninsula and parts of insular Southeast Asia. By the 2090s, particularly under SSP3-7.0 and SSP5-8.5, contraction also became more evident in southern and central China. Newly suitable areas occurred mainly in northern China, Mongolia, southern Siberia and the Russian Far East, but these gains were smaller than the losses across lower-latitude regions.

The strongest contrast between the two species occurred across South and Southeast Asia, where expansion of *R. pedestris* broadly coincided with contraction of *N. viridula*. Overall, *R. pedestris* retained extensive stable suitable areas and expanded mainly across northern Asia, whereas *N. viridula* lost large portions of its suitable area in South and Southeast Asia and became increasingly concentrated in East Asia.

Consistent with these spatial patterns, the total suitable area of *R. pedestris* increased from 12.599 million km^2^ under current conditions to 14.904–15.386 million km^2^ in the 2050s and 15.031–16.888 million km^2^ in the 2090s, corresponding to increases of 18.3–34.0%. In contrast, the total suitable area of *N. viridula* declined from 12.430 million km^2^ to 9.883–11.081 million km^2^ in the 2050s and 9.108–10.970 million km^2^ in the 2090s, representing decreases of 10.9–26.7%.

### 3.4. Reorganization of Two-Species Suitable-Area Overlap

**Figure 3:**
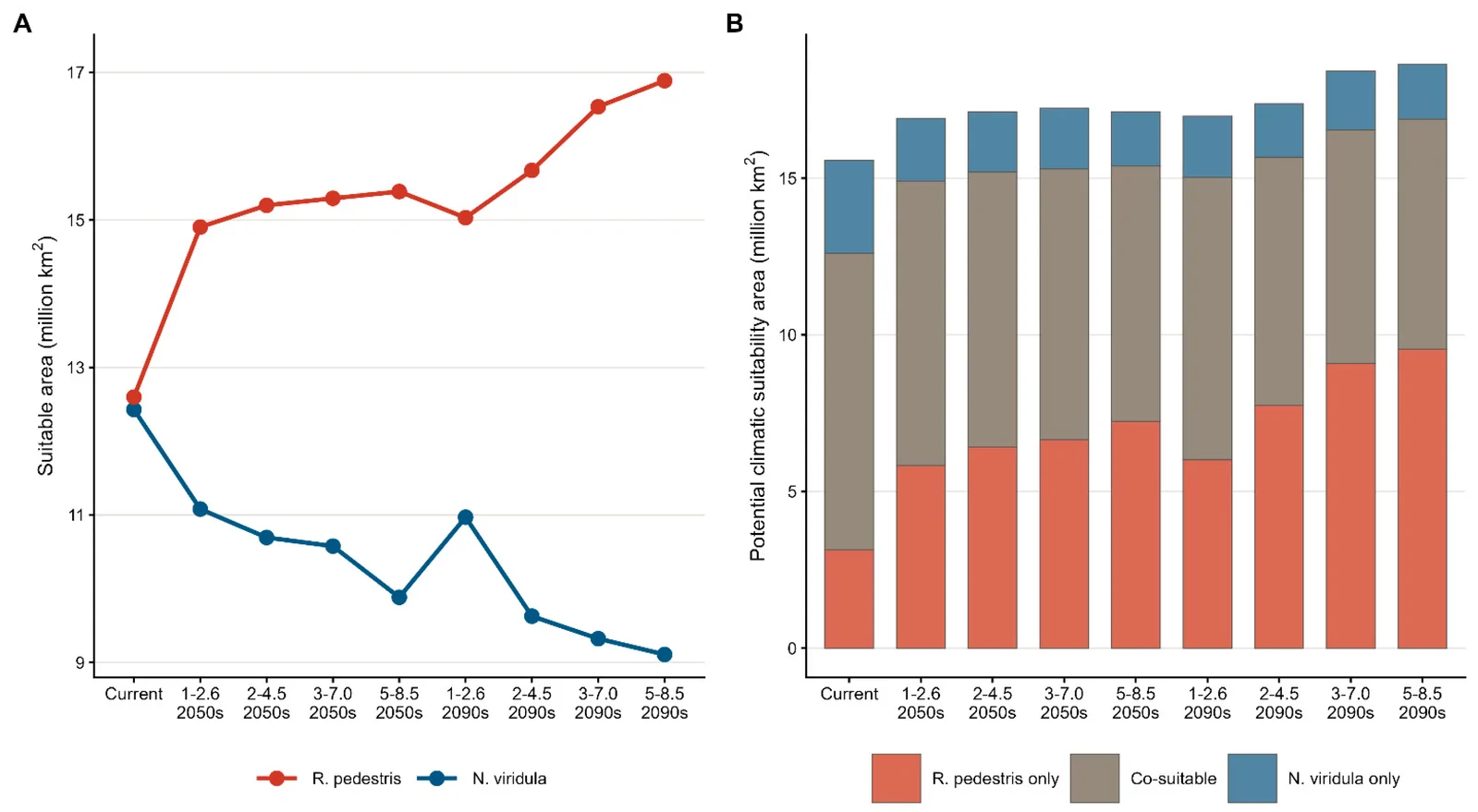
Suitable-area changes and two-species overlap. (**A**) Total suitable area under current and future scenarios. (**B**) Co-suitable, *R. pedestris*-only and *N. viridula*-only areas.

The overlap analysis partitioned the Asian analysis extent into four categories: co-suitable areas, areas suitable exclusively for *R. pedestris*, areas suitable exclusively for *N. viridula*, and areas unsuitable for both species (Figure 3B; Figure S10; Table 3).

Under current climatic conditions, co-suitable areas formed a broad zone extending from central and eastern China through the Korean Peninsula and Japan, and continued across parts of the Indian subcontinent, mainland Southeast Asia and insular Southeast Asia. *N. viridula*-only suitable areas occurred mainly in western and southern parts of the Indian subcontinent, along the Himalayan region and in parts of Central Asia. In contrast, *R. pedestris*-only suitable areas were concentrated mainly across northern and northeastern Asia, with additional scattered areas in the Indian subcontinent and Southeast Asia.

Under future climate scenarios, the co-suitable zone remained extensive across central and eastern China, the Korean Peninsula and Japan, but gradually contracted across South and Southeast Asia. At the same time, *R. pedestris*-only suitable areas expanded markedly across northern Asia and became increasingly widespread across the Indian subcontinent, mainland Southeast Asia and insular Southeast Asia. Areas suitable only for *N. viridula* became more restricted and fragmented, remaining mainly in western South Asia, the Himalayan region and parts of Central Asia. These changes became most pronounced in the 2090s under SSP3-7.0 and SSP5-8.5.

Quantitatively, the current co-suitable area was 9.460 million km^2^, while *R. pedestris*-only and *N. viridula*-only suitable areas covered 3.139 and 2.970 million km^2^, respectively. In the 2050s, the co-suitable area declined to 8.150–9.073 million km^2^, while *R. pedestris*-only suitable areas increased to 5.831–7.235 million km^2^ and *N. viridula*-only suitable areas decreased to 1.733–2.007 million km^2^. By the 2090s, the co-suitable area ranged from 7.350 to 9.017 million km^2^, *R. pedestris*-only suitable areas increased to 6.014–9.538 million km^2^, and *N. viridula*-only suitable areas ranged from 1.704 to 1.953 million km^2^. Overall, future overlap dynamics were characterized by contraction of the co-suitable area and expansion of *R. pedestris*-only suitable areas, particularly under stronger late-century warming.

### 3.5. Climatic Niche Differentiation in Environmental Space

**Figure 4:**
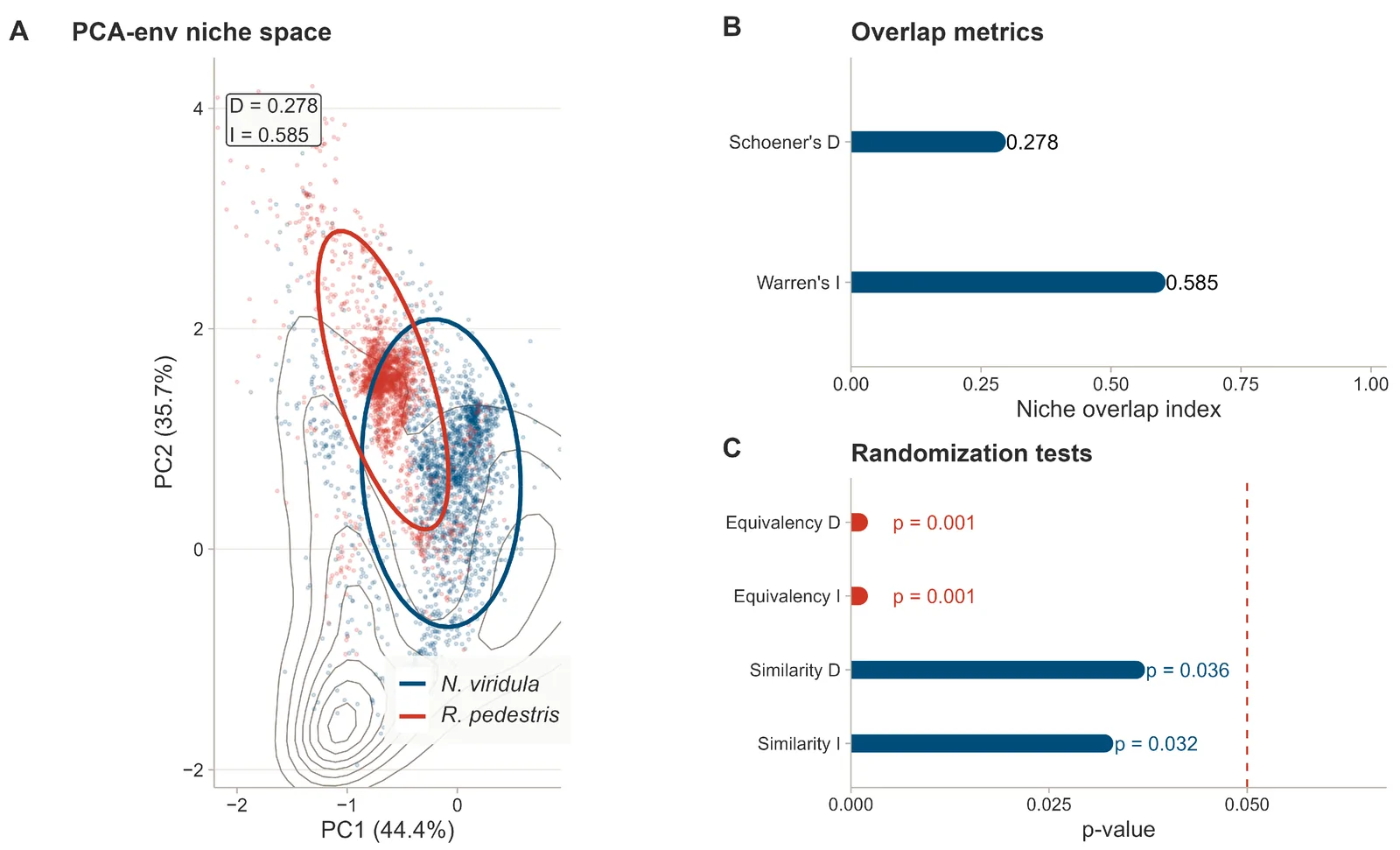
PCA-env climatic niche comparison of *R. pedestris* and *N. viridula*. (**A**) Niche densities in PCA-env space. (**B**) Niche overlap (Schoener’s D and Warren’s I). (**C**) Equivalency and similarity randomization tests. PCA-env used the five variables BIO2, BIO5, BIO6, BIO14, and BIO18 to preserve a shared environmental comparison space.

PCA-env explained 80.1% of environmental variation on the first two axes: PC1 was temperature-related (BIO5 and BIO6 loadings −0.913 and −0.914), whereas PC2 was precipitation-related (BIO14 and BIO18 loadings 0.811 and 0.731) (Figure 4; Figure S6).

Niche overlap was low to moderate (Schoener’s D = 0.278; Warren’s I = 0.585). Equivalency tests were significant (*p* = 0.001 for both indices), rejecting niche equivalency, while similarity tests were also significant (D *p* = 0.036, I *p* = 0.032), indicating overlap greater than background expectation. A subsampling sensitivity test (100 iterations with *N. viridula* randomly reduced to *n* = 1985) confirmed that sample-size asymmetry did not drive these results: median subsampled D = 0.267 (95% interval 0.246–0.294) and median I = 0.537 (95% interval 0.512–0.578), both consistent with the full-data estimates (Table S4).

Dynamic indices indicated high stability but directional environmental differences. With *R. pedestris* as reference and *N. viridula* as focal, expansion, stability and unfilling were 0.063, 0.937 and 0.001; with the reverse reference, they were 0.001, 0.999 and 0.063 (Table S4). The niche of *R. pedestris* was largely nested within that of *N. viridula* (stability ≥ 0.937 in both directions), but density peaks were positioned differently: *R. pedestris* concentrated along the warm-season precipitation axis (BIO18), whereas *N. viridula* spread more broadly along the cold-month temperature axis (BIO6).

### 3.6. Phenological Matching Under Fixed Soybean Calendars

**Figure 5:**
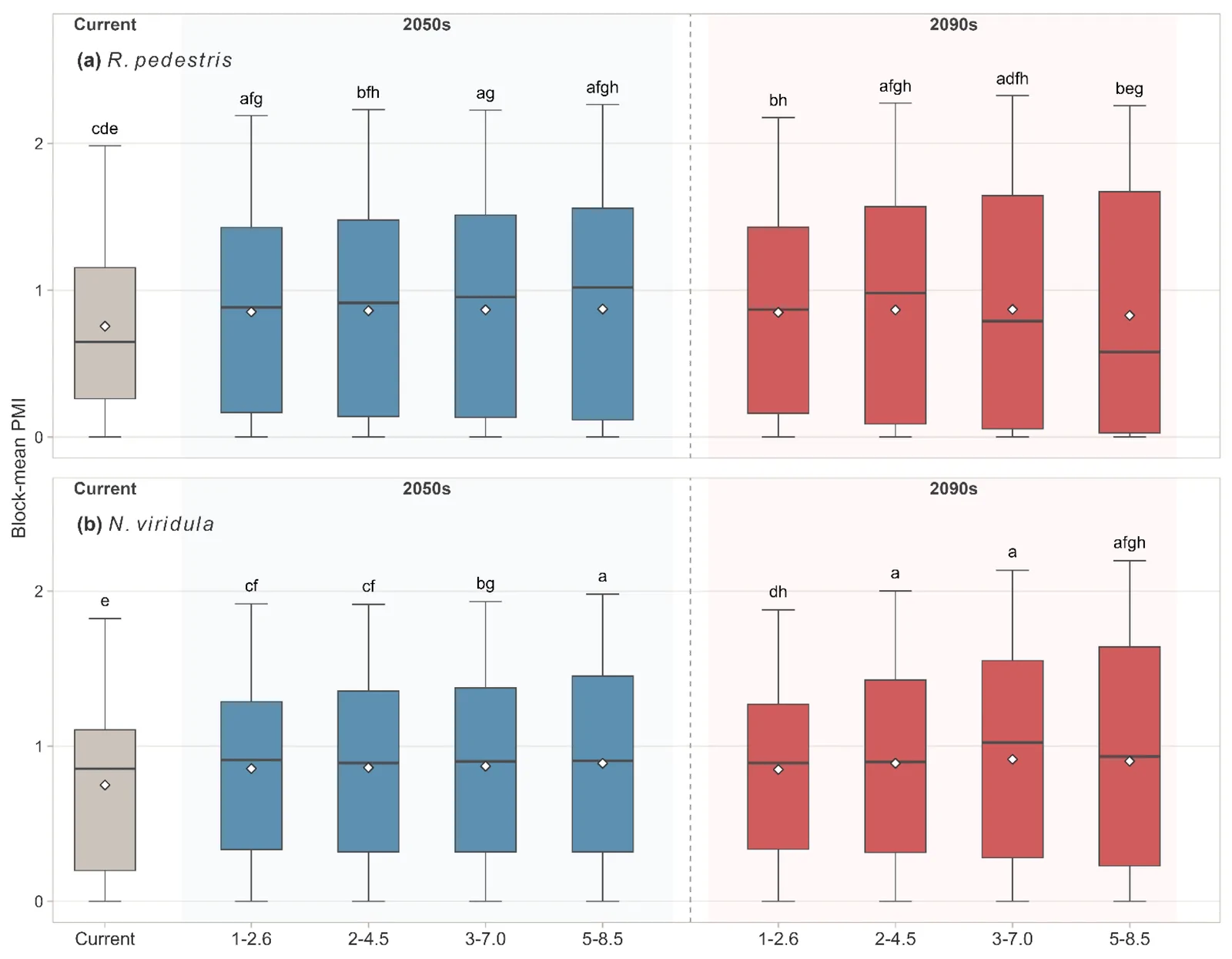
Block-mean PMI for (**a**) *R. pedestris* and (**b**) *N. viridula* under current and future climates within 5° × 5° spatial blocks. The *x*-axis shows one current baseline and eight future scenarios (four SSPs × two periods). Letters indicate compact letter displays from pairwise Wilcoxon signed-rank tests with Benjamini–Hochberg correction; groups sharing a letter are not significantly different (α = 0.05).

Within current soybean-growing regions, the baseline mean PMI was 0.873 for *N. viridula* and 0.964 for *R. pedestris* (Table S5a). Under fixed soybean calendars, future warming generally increased the temporal overlap between potential adult activity windows and soybean R3–R7 growth stages for both pest species (Figure 5).

For *R. pedestris*, the mean PMI increased under all 2050s scenarios and reached its maximum value of 1.136 under SSP3-7.0 in the 2050s. These increases were statistically significant relative to the baseline across all 2050s scenarios. By the 2090s, however, the magnitude of increase weakened under stronger warming. The mean PMI declined to 1.036 under SSP5-8.5 in the 2090s, and the increase relative to the baseline was no longer statistically significant under SSP3-7.0 or SSP5-8.5 in the 2090s.

For *N. viridula*, the mean PMI increased under all future scenarios and remained significantly higher than the baseline after Benjamini–Hochberg correction. The highest value was observed under SSP3-7.0 in the 2090s, reaching 1.130. Unlike *R. pedestris*, *N. viridula* did not exhibit a clear late-century attenuation under high-emission scenarios.

Overall, fixed-calendar PMI increased for both pest species, indicating enhanced temporal overlap between potential adult activity and soybean reproductive stages under future climates. However, the two species showed distinct trajectories. *R. pedestris* exhibited a mid-century increase followed by attenuation under late-century high-emission scenarios, whereas *N. viridula* showed a more persistent increase across future climate scenarios.

### 3.7. Effects of Sowing-Calendar Adjustment on Phenological Matching

**Figure 6:**
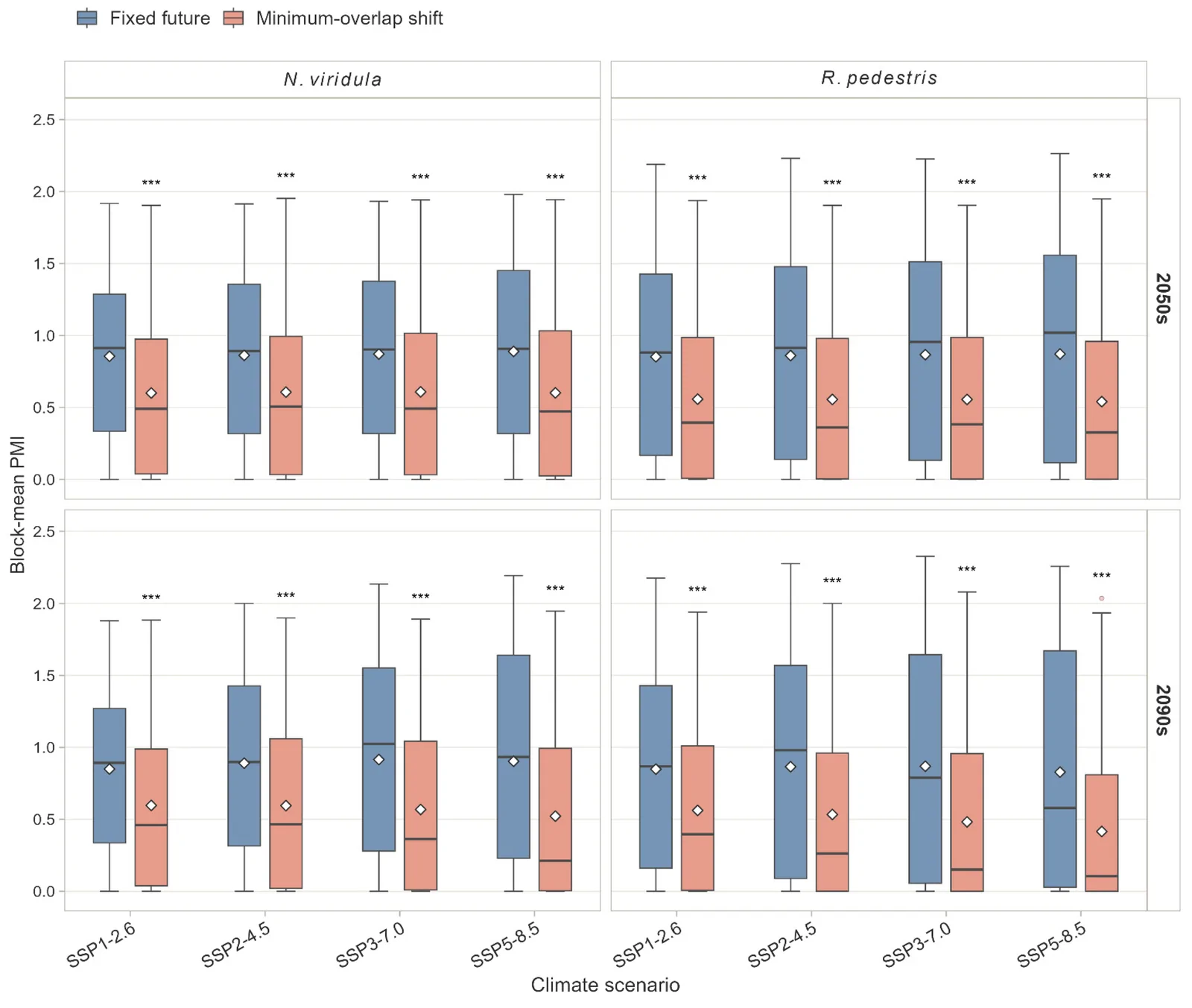
Effect of sowing-date adjustment on PMI. Block-mean PMI under fixed future calendars (blue) and the minimum-overlap 30 d sowing delay (salmon). Asterisks denote significant differences between the two calendars (paired Wilcoxon signed-rank test, Benjamini–Hochberg-adjusted: (*** *p* < 0.001).

Among the four discrete sowing-date scenarios tested as exposure-screening experiments, a 30 d sowing delay (+30 d) yielded the lowest mean PMI for every species, SSP and period (Figure 6; Figure S8; Table S5b). Advancing sowing instead raised exposure in the warmest scenarios; for example, for *R. pedestris* under the 2090s SSP5-8.5, a 30 d advance increased mean PMI from 1.036 to 1.306, consistent with adult-active windows having shifted earlier under warming.

Compared with fixed future calendars, the 30 d delay reduced mean PMI by 0.26–0.47 for *N. viridula* and 0.38–0.55 for *R. pedestris*, with significant reductions in all scenarios (Figure 6; Figure S9; Table S5b). Under the minimum-overlap scenario, mean PMI declined to 0.63–0.79 for *N. viridula* and 0.48–0.73 for *R. pedestris*.

Window-length sensitivity analysis showed that changing the adult-active window to 25 d or 35 d altered absolute PMI values but preserved spatial rankings (Spearman ≥ 0.994; Figure S7; Table S5c).

## 4. Discussion

### 4.1. Species-Specific Reorganization of Climatic Suitability Under Warming

Future projections revealed contrasting spatial trajectories for the two soybean pests. *Riptortus pedestris* retained its core suitable areas in temperate East Asia and exhibited a net expansion, with newly suitable areas occurring primarily across northern, northeastern, and inland Asia. In contrast, the projected gains of *Nezara viridula* at higher latitudes were insufficient to offset its extensive losses across South and Southeast Asia, resulting in a net contraction and an increasing concentration of suitable areas in temperate East Asia. Accordingly, the co-suitable area declined, whereas the area suitable only for *R. pedestris* increased substantially.

The projected expansion of *R. pedestris* is consistent with an earlier MaxEnt study that predicted a marked increase in its future suitable range, with new areas of high suitability occurring mainly in northeastern China and western India [9]. For *N. viridula*, the projected gains in temperate East Asia are consistent with its documented northward range expansion in Japan [11,12]. Its projected losses at lower latitudes also broadly accord with the long-term decline in the relative abundance and pest status of *N. viridula* reported in agricultural systems in the Neotropical region [13]. More broadly, the concurrent expansion at higher latitudes and contraction in warmer regions is consistent with evidence that climate-related distributional changes may differ between the leading and trailing margins of pest ranges [45,46]. Taken together, the two species showed distinct modeled responses to future warming: *R. pedestris* was characterized by broad net expansion, whereas *N. viridula* exhibited a spatially asymmetric response, with limited gains at higher latitudes outweighed by extensive losses across its warmer, low-latitude range.

Both pests can adversely affect soybean production. Feeding by *R. pedestris* reduces seed quality and germination and is closely associated with soybean staygreen syndrome [2,3], whereas *N. viridula* causes seed damage, yield loss, and delayed crop maturity [4,5,17]. Under current climatic conditions, the co-suitable zone overlaps major soybean-producing regions in temperate East Asia, suggesting that both pests should remain management considerations in these established production areas. Under future climates, the expansion of *R. pedestris* toward northern and northeastern Asia may extend climatically suitable conditions for this pest across a broader portion of northern soybean-growing regions. By contrast, despite its overall contraction, *N. viridula* is projected to retain a core suitable area in temperate East Asia. Both species should therefore remain within the scope of pest surveillance and management in the current co-suitable regions, whereas northern and northeastern soybean-producing regions may increasingly require greater attention to *R. pedestris*.

### 4.2. Climatic Associations and Niche Differentiation

Previous field and laboratory studies indicate that rainfall and temperature are associated with the seasonal abundance and population performance of *R. pedestris*. Field monitoring in Chungbuk Province reported that seasonal fluctuations in *R. pedestris* abundance in 2011 were closely associated with environmental conditions, particularly rainfall [47]. Experimental life-table studies further showed that the development, survival, reproduction, and population growth of *R. pedestris* varied with temperature, with overall population performance generally being highest at intermediate temperatures [42,48,49]. Consistent with these field and laboratory observations, precipitation of the warmest quarter (BIO18) was the most important predictor in the present model, whereas isothermality (BIO3) and maximum temperature of the warmest month (BIO5) provided additional predictive information. Warm-season moisture and seasonal thermal regimes may therefore represent important dimensions of the climatic space occupied by *R. pedestris*.

For *N. viridula*, previous field studies linked its northern range limit in Japan to winter temperature conditions and documented a progressive northward range expansion [11,12,50]. Laboratory experiments revealed a nonlinear developmental response to temperature: development was fastest at intermediate temperatures, whereas the species failed to complete development at either 15 or 35 °C [43]. Further experiments showed that elevated summer temperatures reduced growth, survival, and reproductive performance [51], while exposure to high temperatures disrupted the obligate gut symbiosis required for normal development [52]. In the present model, minimum temperature of the coldest month (BIO6) was the dominant predictor, followed by maximum temperature of the warmest month (BIO5). Together, the relative importance of BIO6 and BIO5 suggests that both winter minimum and summer maximum temperatures are relevant to the modeled climatic suitability of *N. viridula*.

Comparative niche studies have shown that agricultural pests sharing hosts or geographic ranges can nevertheless occupy distinct climatic spaces. Within the *Bemisia tabaci* species complex, invasive and native cryptic species differed in niche breadth, environmental associations, and the degree of niche overlap [53]. Similarly, comparative analyses of the maize pests *Ostrinia furnacalis* and *O. nubilalis* identified differences in climatic niche occupancy and projected niche dynamics [54]. In the present study, PCA-env analysis showed that the climatic niches of *R. pedestris* and *N. viridula* overlapped partially but were not equivalent. *R. pedestris* was more strongly associated with the precipitation axis, whereas *N. viridula* was more strongly associated with the temperature axis. Taken together, the MaxEnt and PCA-env results indicate that the two pests occupy partially overlapping but climatically differentiated environmental spaces, a pattern consistent with their contrasting current distributions and projected future spatial responses.

### 4.3. Species-Specific Phenological Matching and Crop-Calendar Effects

In wheat systems, warming advanced the eclosion of overwintering *Helicoverpa armigera* and increased early-season larval infestation of younger, more susceptible plants, thereby increasing yield loss [55]. Similarly, crop–pest simulations for maize–soybean systems in the American Midwest projected earlier pest activity and prolonged crop exposure to damaging pest stages under future warming [56]. Together, these studies indicate that warming-induced shifts in pest phenology may intensify crop damage when damaging stages coincide more closely with susceptible crop growth periods. Accordingly, the present study evaluated whether future warming could alter the overlap between the potential adult activity windows of *R. pedestris* and *N. viridula* and soybean R3–R7.

PMI projections revealed species-specific changes in phenological overlap. Under fixed soybean calendars, PMI increased under all future scenarios for *N. viridula*. For *R. pedestris*, PMI increased significantly under all four scenarios in the 2050s; however, the increase was smaller in the 2090s and was no longer significant under SSP3-7.0 and SSP5-8.5. These projections suggest that warming may lead to a more persistent increase in the overlap between the potential adult activity of *N. viridula* and soybean R3–R7, whereas the corresponding increase for *R. pedestris* may be concentrated mainly in the 2050s and weaken by the 2090s.

In southern Japan, delayed sowing reduced soybean bug densities and seed damage [57]. In Mississippi, stink bug populations peaked from early to mid-September. Early planted soybean had already reached physiological maturity before this peak, whereas later-planted maturity group V soybean remained at R6–R7, harbored the highest stink bug densities, and exceeded the local economic threshold during the peak period [58]. Similarly, in northern Ghana, early-planted soybean matured before the late-season peak of a pod-sucking bug complex dominated by *N. viridula*, and was associated with lower pest infestation and seed damage and higher yields than later plantings [59]. Collectively, these studies indicate that sowing date can alter the timing of soybean reproductive development relative to seasonal stink bug peaks, with consequences for pest infestation, crop damage, and yield. In the present scenario analysis, a 30-day sowing delay produced the lowest PMI for both species under every future climate combination. This result indicates that, within the soybean calendars evaluated here, sowing-date adjustment may partially offset projected warming-induced increases in phenological overlap between soybean R3–R7 and the potential adult activity windows of both pests.

### 4.4. Scope and Limitations

The MaxEnt projections represent potential climatic suitability inferred from associations between presence-only occurrence records and climatic predictors [25,29]. Because MaxEnt is a presence–background modeling approach, its outputs should not be interpreted as direct estimates of species occurrence, population abundance, or soybean damage [28,60]. The projections instead describe broad-scale changes in the extent and spatial distribution of climatically suitable areas. Regional occurrence records and field surveys are therefore required to evaluate the projected patterns of expansion and contraction.

The PMI analysis was designed for broad-scale comparison. Monthly WorldClim temperature layers provided a spatially consistent climatic framework across current and future scenarios [20]. However, monthly aggregation smooths short-term temperature variation, which can influence accumulated degree-days and the estimated timing at which developmental thresholds are reached [61,62]. The crop calendar used in this study represented one soybean growing cycle per grid cell, based on the global planting and harvesting dates compiled by Sacks et al. [40]. PMI therefore applies only to the represented crop cycle and does not include additional soybean seasons in multiple-cropping regions [63].

Adult activity windows were inferred from accumulated temperature using a single set of developmental parameters for each species. This simplification does not account for geographic variation in thermal requirements among insect populations [64] or feeding by late-instar nymphs. Diapause was not explicitly modeled. In both *R. pedestris* and *N. viridula*, diapause is regulated by photoperiod and temperature, and seasonal activity may therefore not begin immediately after the required thermal accumulation has been reached [65,66]. Omitting diapause may consequently lead to overestimation of phenological overlap in some regions [67,68]. PMI should therefore be interpreted as a relative thermal-overlap index between the modeled adult activity windows and soybean R3–R7, rather than as a direct prediction of the actual hazard period. Regional validation will require field observations of pest seasonal activity and soybean phenology.

## 5. Conclusions

Future warming is expected to reshape the climatic suitability of the two soybean pests in markedly different ways. *Riptortus pedestris* is projected to retain its core suitable areas in temperate East Asia and expand across northern, northeastern, and inland Asia. In contrast, *Nezara viridula* is projected to lose extensive suitable areas across South and Southeast Asia, despite limited gains at higher latitudes. As a result, the co-suitable area is expected to decline, whereas areas suitable only for *R. pedestris* are likely to expand.

Under fixed soybean calendars, warming may lead to a persistent increase in phenological overlap between the potential adult activity of *N. viridula* and soybean R3–R7. For *R. pedestris*, the increase may be concentrated mainly in the 2050s and weaken by the 2090s. A 30-day delay in sowing produced the lowest PMI for both species under all future climate combinations, suggesting that adjustment of sowing date may partially mitigate warming-induced increases in phenological overlap. These findings indicate that both pests should remain a concern in soybean-producing regions of temperate East Asia, whereas northern and northeastern production regions may require increasing attention to *R. pedestris*. Regional field observations of pest seasonal activity and soybean phenology will be necessary to evaluate these projected spatial and temporal changes.

## Figures and Tables

**Table 1:** Species-specific MaxEnt model configuration, validation performance and FCV5 suitability thresholds for *R. pedestris* and *N. viridula*.

Species	Occurrences	Background	Species-Specific Predictors	FC/RM	AICc	Validation AUC/Evaluation TSS/Validation CBI	FCV5 Threshold	Top Predictor
*R. pedestris*	1985	10,000	BIO18, BIO5, BIO14, BIO2, BIO3, BIO12	LQHPT/2.0	14,453.349	0.980/0.912/0.820	0.023	BIO18 (63.3%)
*N. viridula*	21,160	10,000	BIO6, BIO5, BIO14, BIO18, BIO2, BIO15, BIO19, BIO13, BIO8, BIO9	LQHP/0.5	14,893.407	0.958/0.835/0.953	0.098	BIO6 (66.6%)

Note: FC = feature class; RM = regularization multiplier; CBI = continuous Boyce index. AUC and CBI are mean block-validation metrics; TSS is the full-fit evaluation value at MTSPS. FCV5 is the species-specific threshold used for suitability mapping. Both final models used the same 10,000 global background coordinates.

**Table 2:** Current and future suitable area (million km^2^) for *R. pedestris* and *N. viridula* within the Asian analysis extent. Suitable area is the sum of low, medium, and high suitability classes.

Species	Period	Scenario	Total Suitable Area (Million km^2^)	High Suitability Area (Million km^2^)	Change from Current (Million km^2^)	Change from Current (%)
*Nezara viridula*	Current	Current	12.430	3.016	0.000	0.0
	2050s	SSP1-2.6	11.081	2.301	−1.350	−10.9
	2050s	SSP2-4.5	10.694	2.361	−1.736	−14.0
	2050s	SSP3-7.0	10.578	2.230	−1.853	−14.9
	2050s	SSP5-8.5	9.883	1.994	−2.547	−20.5
	2090s	SSP1-2.6	10.970	2.408	−1.460	−11.7
	2090s	SSP2-4.5	9.628	1.774	−2.802	−22.5
	2090s	SSP3-7.0	9.323	1.327	−3.107	−25.0
	2090s	SSP5-8.5	9.108	0.883	−3.322	−26.7
*Riptortus pedestris*	Current	Current	12.599	3.481	0.000	0.0
	2050s	SSP1-2.6	14.904	4.374	2.305	18.3
	2050s	SSP2-4.5	15.198	4.495	2.599	20.6
	2050s	SSP3-7.0	15.294	4.424	2.694	21.4
	2050s	SSP5-8.5	15.386	4.640	2.786	22.1
	2090s	SSP1-2.6	15.031	4.472	2.431	19.3
	2090s	SSP2-4.5	15.672	4.677	3.073	24.4
	2090s	SSP3-7.0	16.535	5.165	3.936	31.2
	2090s	SSP5-8.5	16.888	5.451	4.289	34.0

**Table 3:** Two-species climatic suitability overlap (million km^2^) within the Asian analysis extent.

Period	Scenario	Co-Suitable (Million km^2^)	*Nezara viridula* Only (Million km^2^)	*Riptortus pedestris* Only (Million km^2^)
Current	Current	9.460	2.970	3.139
2050s	SSP1-2.6	9.073	2.007	5.831
2050s	SSP2-4.5	8.775	1.919	6.423
2050s	SSP3-7.0	8.640	1.937	6.653
2050s	SSP5-8.5	8.150	1.733	7.235
2090s	SSP1-2.6	9.017	1.953	6.014
2090s	SSP2-4.5	7.923	1.704	7.749
2090s	SSP3-7.0	7.441	1.882	9.094
2090s	SSP5-8.5	7.350	1.758	9.538

## Data Availability

The cleaned occurrence records, full PMI sowing-date scenario data and analysis scripts supporting this study are archived in Zenodo (10.5281/zenodo.20536989). GBIF occurrence data were downloaded on 23 March 2026 (*R. pedestris*: https://doi.org/10.15468/dl.9prazt; *N. viridula*: https://doi.org/10.15468/dl.uwm2kv). Current and future bioclimatic variables were obtained from WorldClim v2.1 (https://www.worldclim.org). Soybean physical area data were obtained from SPAM 2020 v2.0 Release 2 (https://doi.org/10.7910/DVN/SWPENT).

## Supplementary Materials

The following supporting information can be downloaded at: https://www.mdpi.com/article/10.3390/insects17080753/s1. Figure S1: Environmental-variable screening for *R. pedestris* and *N. viridula*; Figure S2: MaxEnt model tuning and performance evaluation for *R. pedestris* and *N. viridula*; Figure S3: Marginal response curves of the MaxEnt models for *R. pedestris* and *N. viridula*; Figure S4: Current and future climatic suitability classes across SSPs and projection periods within the Asian analysis extent; Figure S5: Global distribution of cleaned and spatially thinned occurrence records used for MaxEnt modelling; Figure S6: PCA-env environmental space and loadings of the five bioclimatic variables used for niche comparison; Figure S7: Sensitivity of the phenological matching index to adult-activity window length; Figure S8: Spatial patterns of the phenological matching index under five sowing-date shifts across species and SSPs; Figure S9: Rank correlations between shifted-calendar and fixed-future PMI across sowing-date shifts; Figure S10: Spatial reorganization of climatic suitability overlap under current and future climates within the Asian analysis extent; Table S1: Occurrence data, modelling inputs and environmental-variable screening; Table S2: MaxEnt model tuning, performance evaluation and permutation importance; Table S3: FCV5 thresholds, Jenks class boundaries, suitable-area estimates and future suitable-area transitions; Table S4: PCA-env niche comparison: overlap tests, variable loadings, dynamic indices and subsampling sensitivity; Table S5: Phenological matching index analyses: fixed-calendar scenarios, sowing-date shifts, window-length sensitivity, thermal-development parameters and statistical tests.
